# Cluster-based histopathology phenotype representation learning by self-supervised multi-class-token hierarchical ViT

**DOI:** 10.1038/s41598-024-53361-0

**Published:** 2024-02-08

**Authors:** Jiarong Ye, Shivam Kalra, Mohammad Saleh Miri

**Affiliations:** Roche Diagnostics Solutions, Santa Clara, CA USA

**Keywords:** Machine learning, Image processing

## Abstract

Developing a clinical AI model necessitates a significant amount of highly curated and carefully annotated dataset by multiple medical experts, which results in increased development time and costs. Self-supervised learning (SSL) is a method that enables AI models to leverage unlabelled data to acquire domain-specific background knowledge that can enhance their performance on various downstream tasks. In this work, we introduce *CypherViT*, a cluster-based histo-pathology phenotype representation learning by self-supervised multi-class-token hierarchical Vision Transformer (ViT). *CypherViT* is a novel backbone that can be integrated into a SSL pipeline, accommodating both coarse and fine-grained feature learning for histopathological images via a hierarchical feature agglomerative attention module with multiple classification (cls) tokens in ViT. Our qualitative analysis showcases that our approach successfully learns semantically meaningful regions of interest that align with morphological phenotypes. To validate the model, we utilize the DINO self-supervised learning (SSL) framework to train *CypherViT* on a substantial dataset of unlabeled breast cancer histopathological images. This trained model proves to be a generalizable and robust feature extractor for colorectal cancer images. Notably, our model demonstrates promising performance in patch-level tissue phenotyping tasks across four public datasets. The results from our quantitative experiments highlight significant advantages over existing state-of-the-art SSL models and traditional transfer learning methods, such as those relying on ImageNet pre-training.

## Introduction

Access to large-scale and good quality dataset is a primary driver in machine learning, with well-known datatsets such as ImageNet^1^ in computer vision has led to remarkable achievements in the domain of natural images. For medical image analysis tasks, labeled data is scarce and expensive as it requires annotations from multiple experts and crowd-sourcing is generally not an option. Furthermore, inter-observer variability among medical experts affects the quality of the dataset^2^. Due to these reasons, it is both cost and time prohibitive to assemble a large and good-quality dataset for medical imaging analysis tasks which limits the progress of research and model development. Unsupervised machine learning leveraging unlabelled data could provide a solution to these challenges, and promote the development of more accurate AI models. Utilizing a network pre-trained on ImageNet dataset as a starting point is a common practice for model development for medical imaging applications. However, natural-scene images offer vastly different features and patterns than medical images which may limit the model’s ability to converge or prolong its training. A supporting study^3^ has shown that the performance of a large model trained through transfer learning across different domains has equivalent performance to a smaller model trained from scratch.

Histopathology has seen widespread adoption of digitization, offering unique opportunities to increase objectivity and accuracy of diagnostic interpretations through machine learning^2^. Digital images of tissue specimens exhibit significant complexity and heterogeneity from the preparation, fixation, and staining protocols, among other factors. This variety further exacerbates the accessibility to a large labeled dataset in digital pathology as compared with any other medical imaging modalities. Furthermore, each histopathology image is generally a gigapixel file which requires significantly more manual-labeling effort leading to higher inter/intra-observer variability and mis-localization of regions of interest. These challenges strengthen the imperative of utilization of unsupervised machine learning approaches to leverage the vast amounts of unlabelled data in the digital pathology domain.

Self-supervised learning (SSL) is a form of unsupervised learning, designed to learn domain-specific salient features from vast amount of unlabelled data. It is a highly active research field that provides a solution to enable AI models to acquire domain-specific background knowledge from the massive amount of existing unlabeled data. It learns visual representations of images based on supervised signals that are completely derived from the data itself. Various SSL techniques^4–10^ in the literature have been validated on natural images that are already adopted as industry standard practices, but their value-proposition has not yet been realized or explored for the digital pathology algorithm development. There is a need for adaptation of the existing SSL methods to histopathology data that offers vastly different characteristics than natural images (e.g. features such as cell density, cell morphology, etc are not present in natural images). SSL allows AI models to discover domain-specific background knowledge about the data without requiring labels from subject matter experts. This means the high-level general knowledge of the field is learned from the unlabeled data making it easier to learn task-specific information/skills (such as cell segmentation, screening for tumor type, etc) in a supervised manner even when limited labeled data is available. SSL significantly reduces the dependency on the accessibility of large labeled dataset for developing new clinical algorithms, thus promoting opportunities for model development. The generalization gap in a clinical AI algorithm is usually larger when it is trained on a limited amount of data due to limited diversity. SSL can bridge this gap by building more generalist models that act as a better starting point for training on specific downstream tasks than training from the scratch.

**Figure 1:**
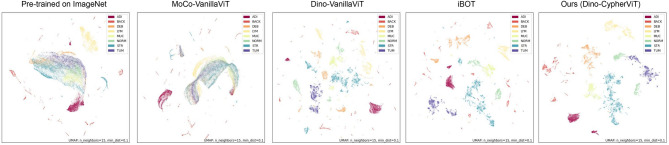
2D UMAP visualization of feature embeddings extracted from models pre-trained on ImageNet, existing state-of-the-art SSL models (MoCo-VanillaViT^4^, DINO-VanillaViT^10^, IBOT^11^), and our proposed DINO-*CypherViT* pre-trained on VGH dataset^12^. The feature embeddings are obtained by employing SSL backbone encoders on the CRC dataset w/ normalization. We follow the default parameters setting for UMAP plotting (neighbors = 15, dist = 0.1). As we can observe, feature embeddings from DINO with the proposed *CypherViT* backbone present cleaner and much less noisy clustering results than other state-of-the-art SSL approaches. DINO-CypherViT performs better in terms of concentration and intra- and inter-clustering.

**Figure 2:**
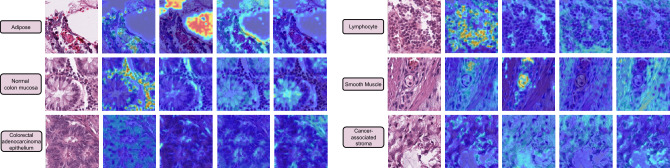
Attention maps extracted from the learnable multi-cls tokens at the final stage of our proposed *CypherViT* (see reference in Figs. 3 and 4). We show the regions of interest highlighted based on relatively high attention scores of 6 tissue types from the CRC dataset w/ normalization (see “Downstream tasks—patch-level tissue phenotyping”) *(Note: here we omit 3 types because there are basically no cells in them: background, debris, and mucus)*. As we can observe, the semantic clustering block can learn semantically interpretable features corresponding to distinct morphological phenotypes.

The main focus of this work is to introduce a novel backbone network for self-supervised learning (SSL) in the field of digital histopathology images. With the increasing availability of unlabeled digital tissue data due to rapid digitization, our proposed SSL technique aims to leverage this data to enhance the robustness of data-intensive AI-based clinical algorithms while reducing development costs. Our contributions can be summarized as follows:

1. We present *CypherViT*, a cluster-based histopathology phenotype representation learning model integrated as a backbone network within the DINO^10^ SSL framework. This innovative backbone captures both coarse and fine-grained features, providing advantages over existing approaches. Specifically, *CypherViT* demonstrates improved feature discrimination, as evidenced by UMAP visualization (Fig. 1). Additionally, it enables precise identification of morphological phenotypes at the pixel level, surpassing the grid structure attention map derived from multi-head attention in ViT, as depicted in Fig. 2.
2. We comprehensively validate the robustness and transferability of our proposed backbone network. Our findings demonstrate successful pattern learning during SSL training on a histopathological dataset of breast cancer, which translates effectively to the evaluation of colorectal cancer images. Moreover, our *CypherViT* backbone with DINO SSL outperforms existing state-of-the-art SSL models in various downstream tasks, including unsupervised and semi-supervised tile classification of tissue types, as well as fine-grained classification of cytological features.
3. Moreover, our proposed *CypherViT* is a SSL framework agnostic backbone that seamlessly integrates into different contrastive-based SSL pipelines like DINO, MOCO, and SimCLR. We have conducted comprehensive experiments to demonstrate its consistent performance improvement compared to the vanillaViT backbone, as shown in Table 3. Its “plug-and-play” capability allows for easy and efficient framework adaptation without significant architectural modifications.

In summary, our work introduces *CypherViT* as a new backbone network specifically designed for SSL in the context of digital histopathology images. By integrating *CypherViT* into the various SSL frameworks, we enhance their feature learning and improve phenotype representation, thereby contributing to the advancement of AI-based clinical algorithms for histopathological analysis.

## Related work

### Contrastive learning in SSL pretext task design

Widely applied in natural image domains, contrastive-based SSL has been proven to be superior in learning remarkable representations^4–6,8,9,13–15^. Contrastive-based approaches pull positive samples closer and repel negative ones, aiming at capturing augmentation-invariant features through a Noise Contrastive Estimation objective^16,17^. In existing works, the contrastive pairs can be sampled from a memory bank as introduced in MoCo^4^, or just from the current batch of data as in SimCLR^5^ when the batch size is sufficiently large. However, there are two downsides to the aforementioned contrastive methods. (i) Firstly, using large batch size in training is computationally expensive. (ii) Secondly, since patches sampled from different spatial locations could be semantically connected therefore may result in false repulsion. To address these limitations, DINO^18^ adopts self-distillation in an unsupervised manner, with centering and sharpening of the momentum teacher outputs to avoid trivial solutions.

### Masked image modeling in SSL pretext task design

Besides using contrastive learning approaches, *Masked Image Modeling (MIM)* is an emerging pretext task first proposed in BEiT^19^. MIM applies block-wise random masking on discrete^19^ or continuous^20,21^ patch tokens and then considers recovering the masked patch tokens or pixels as an auxiliary task. Inspired by the idea of self-distillation used in^8^ and DINO, iBOT^11^ extends MIM by applying loss densely on masked tokens, introducing a hybrid paradigm that combines contrastive learning and MIM to some extent.

### SSL in digital pathology

Besides domain-agnostic applications^22^, to incorporate histopathology-specific knowledge into current frameworks such as SimCLR and MoCo, researchers have proposed hybrid methods to combine contrastive learning and domain-specific pretext tasks designed according to the characteristics of the histopathological images, like predicting magnification levels^23–25^, predicting hematoxylin channel^24^, predicting cross-stain^26^ and normalization^27,28^.

## Results

### SSL training

#### VGH dataset

The dataset^12^ we used for SSL training is the H&E breast cancer dataset built from the Netherlands Cancer Institute (NKI) cohort and the Vancouver General Hospital (VGH) cohort. Patches with less than 70% tissue coverage are filtered out. Patches are cropped to smaller sizes of 224 × 224 pixels from the original resolution of 1128 × 720 pixels, with up to 50% overlap. The dataset is also augmented by applying transformations involving rotations of 90, and 180°, and vertical and horizontal inversion. In total, the post-processed dataset contains ~ 300,000 images.

### Downstream tasks—patch-level tissue phenotyping

#### CRC w/ and w/o Macenko normalization^29^

As training set in downstream tasks, CRC (both w/ and w/o Macenko normalization) include 100,000 hematoxylins & eosin (H &E) stained 224 × 224 histological patches at 20× magnification of human colorectal cancer (CRC) and normal tissue manually extracted from 86 slides. Each image is annotated with a type of tissue label (adipose (Adi), background (Back), debris (Deb), lymphocytes (Lym), mucus (Muc), smooth muscle (Mus), normal colon mucosa (Norm), cancer-associated stroma (Str), colorectal adenocarcinoma epithelium (Tum) ).

#### BreastPathQ dataset^30^

BreastPathQ is a more challenging dataset with noisy and fine-grained labels. We have a training/validation set of 2579/187 patches extracted from 96 H &E slides at 20× magnification with residual invasive breast cancer from the TCGA-BRCA cohort measuring tumor cellularity, i,e. the fractional occupancy of tumor cell presence in the image patch. Each patch has been assigned a tumor cellularity score on a continuous scale from 0 to 1. We report the mean-squared error (MSE) using linear regression and Kendall-Tau concordance.

#### PanNuke dataset^31,32^

PanNuke dataset contains semi-automatically generated nuclei instance images with exhaustive nuclei labels across 19 different tissue types sampled from more than 20K whole slide images at different magnifications, from multiple data sources. In total the dataset contains 205,343 labeled nuclei, each with an instance segmentation mask. But we only use labels in our experiments.

### Evaluation protocol and result analysis

For evaluation, we employ standard protocols on 4 datasets introduced in “Downstream tasks—patch-level tissue phenotyping” by either using frozen features or finetuning the features. We train a k-nearest neighbor (k-NN) classifier and a linear classifier (linear probing) on frozen features extracted from a pre-trained SSL backbone by sweeping over different numbers of nearest neighbors for KNN and different learning rates for linear probing. The results are reported in Table 1. Furthermore, we initialize networks with the pre-trained weights to conduct a semi-supervised experiment using different percentages of annotated images evenly distributed to each class in the training set, while the testing data remains the same as the official splitting. In Table 2, we examine the performance variation of the model trained with 1%, 5%, 10%, 20%, 50%, and 100% of CRC^29^ w/ Macenko normalization. For fair comparison, we implement other existing state-of-the-art self-supervised methods with the same architecture (ViT-small) following the default hyper-parameters setting in their official released codebases and train on the VGH dataset. The evaluation results and ablation studies are shown in Figs. 1, 2 and all tables below, with the best highlighted in purple color. We have some interesting discoveries.

**Table 1:** Top 1 KNN accuracy and linear probing accuracy on patch-level tissue type classification evaluated on CRC dataset w/ and w/o normalization and PanNuke dataset.

Method	CRC (w/o norm)		CRC (w/norm)		PanNuke	BreastPathQ	
	Acc$\uparrow$					MSE$\downarrow$	Tau$\uparrow$
	KNN	Linear	KNN	Linear	KNN	–	
Pretrained on ImageNet	77.99	85.50	82.29	87.03	79.78	0.126	0.357
SimCLR	80.44	85.65	88.25	88.77	82.86	0.049	0.510
MoCo	83.73	85.96	84.51	87.77	81.36	0.198	0.278
iBOT	84.57	87.42	91.48	92.67	90.09	0.031	0.620
DINO	84.01	86.31	90.38	91.42	89.46	0.038	0.608
DINO-*CypherViT*	**89.05**	**90.67**	**93.37**	**94.47**	**93.67**	**0.021**	**0.690**

MSE and Kendall-Tau concordance score on BreastPathQ dataset. The best results among different hyper-parameter settings (see Table 4) are reported here for our model. All SSL methods are using the vanilla ViT backbone except *DINO-CypherViT* which uses our proposed CypherViT backbone.

Significant values are in bold.

**Table 2:** Semi-supervised learning accuracy on patch-level tissue type classification with different percentages of labeled data in CRC train dataset.

Method	CRC (w/normalization)					
	1%	5%	10%	20%	50%	100%
Fully-supervised	51.64	74.04	86.50	88.94	90.66	91.87
MoCo	73.32	88.30	90.18	90.19	91.07	92.05
iBOT	73.36	90.56	91.91	92.20	92.34	92.84
DINO	72.42	89.61	92.05	92.20	93.48	93.76
DINO-*CypherViT*	**76.36**	**91.10**	**93.11**	**93.20**	**93.98**	**94.82**

Addition of 5% labeled data can provide comparable results to the performance using the entire training dataset, which is surpassed when increasing labeled data to 10%. It suggests promising SSL applications to achieve comparable or even better performance via training on much fewer data. All SSL methods are using the Vanilla ViT backbone except *DINO-CypherViT* which uses our proposed CypherViT backbone.

Significant values are in bold.

#### Features are more robust with proper number of local view from multi-crop augmentation depending on tasks

To study the contribution of key hyper-parameters used in the main architecture of *CypherViT* and significant technique to stabilize training, we conduct ablation studies in Table 4, testing performance variations from different combinations of [cls] token number at the final stage and number of local views. And we discover that more local views are beneficial for tasks requiring learned features on a more fine-grained level, such as the calculation of MSE error between linear regression outcome with annotations from BreastPathQ that measures tumor cellularity. While on coarse-grain level, or global features for classification tasks, fewer local views are preferred. For our experiments carried out in Table 4, using 4 [cls] tokens at final stage plus 2 local views from muti-crop augmentation appears to be the most optimal hyper-parameter combination.

#### Semantically meaningful attention maps from multi-[cls] tokens suggest promising interpretability

**Figure 3:**
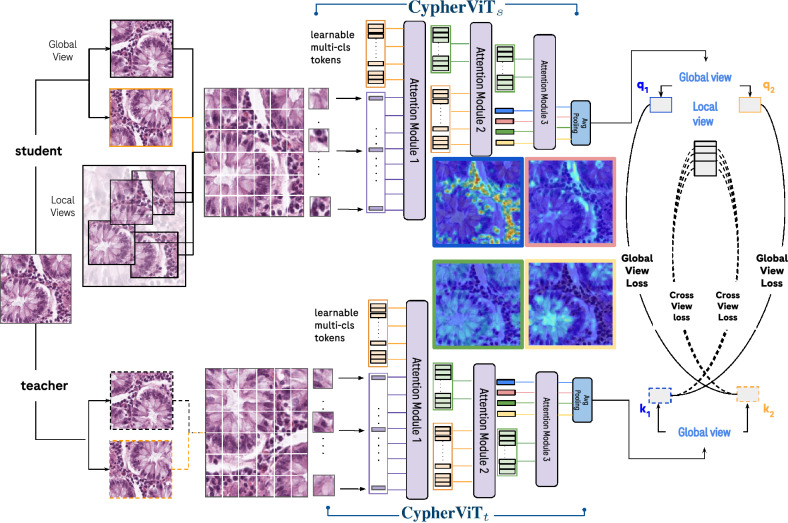
The DINO-*CypherViT* framework. A novel backbone *CypherViT* is used in both student and teacher networks. The *CypherViT* architecture follow the scheme of unsupervised clustering and expand the single-[cls] token in a regular ViT to a set containing learnable multi-[cls] tokens. This set of multi-[cls] tokens assembles coarse to fine-grained features into semantically-aware clusters in a hierarchical manner. As indicated from attention maps visualization from the multi-[cls] tokens before average pooling, the proposed *CypherViT* captures semantically meaningful fine-grained regions of interest detailed to the pixel level during SSL training.

**Figure 4:**
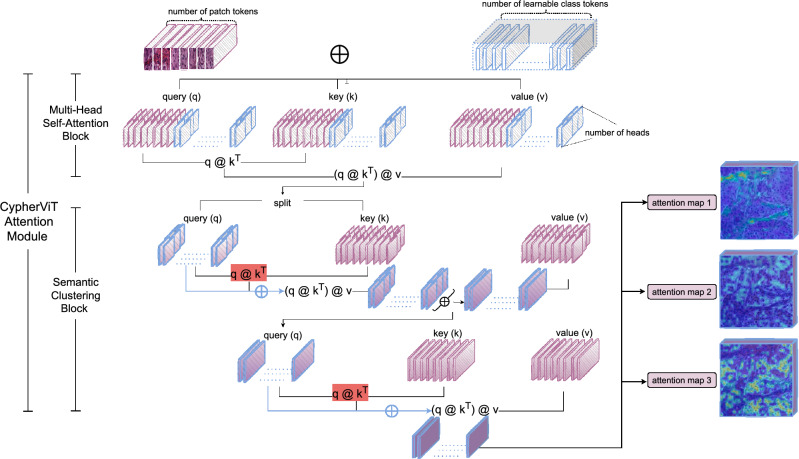
The detailed architecture demonstration of the attention module (*note: * at stage 0) in *CypherViT* (see purple blocks in Fig. 3 for reference).

To provide a more intuitive visualization of what *CypherViT* has learned from SSL training, we overlay the original image with attention maps after interpolating the attention weights extracted from each [cls] token at the final stage of semantic clustering blocks (see reference in Algorithm 1, Figs. 3 and 4). Interestingly, we observe in Fig. 2 that the regions of interest highlighted from learned attentions indicate morphological phenotypes, such as cell in the first column, white space in the second column, and stroma tissue in the last two columns in each class shown. Compared to state-of-the-art self-supervised ViT models such as DINO, ours has two advantages regarding interpretability. Firstly, our attention map presents more fine-grained and precise details in contrast to previous SSL models that provide attention maps in grid structure constrained within regular shape; secondly, our design has more developmental potential to accommodate other machine learning settings for future work. To elaborate, similar to unsupervised clustering, although what each [cls] token actually learns is unknown, it can, however, be easily tweaked in a weakly-supervised experiment setting by slight modification on the loss to each [cls] token to control what each [cls] token should focus on based on what we assign.

**Algorithm 1:**
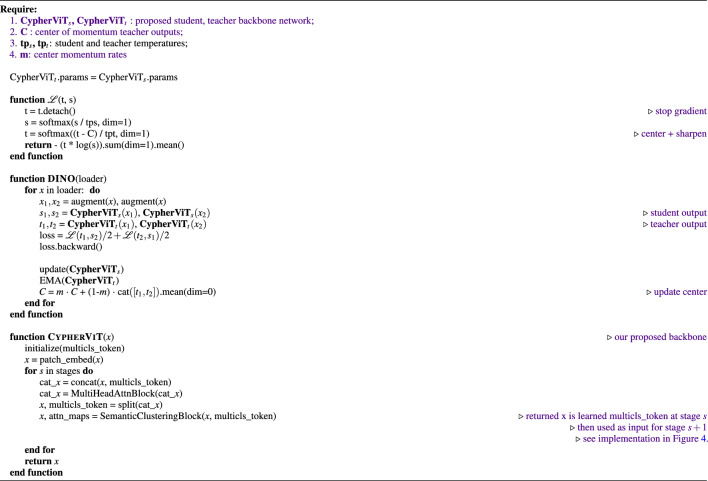
DINO-*CypherViT* pseudocode.

In the latest addition, we aimed to validate the versatility and adaptability of our newly proposed backbone network, CypherViT, by demonstrating its plug-and-play capability across various Self-Supervised Learning (SSL) frameworks beyond its initial integration with DINO. To achieve this, we conducted a thorough ablation study comparing CypherViT with the baseline model, VanillaViT. To ensure the robustness and reliability of our findings, we meticulously combined the official train and test splits of the evaluation dataset. We then proceeded to re-split the data, adhering strictly to the original distribution ratios. This process was repeated five times, each time applying k-NN classification to assess performance. The consistency and stability of the improvements offered by CypherViT were quantitatively showcased in Table 3, where we reported the classification accuracy as an average complemented by the standard deviation. To conclusively demonstrate the performance superiority of our CypherViT over the baseline VanillaViT, we conducted t-tests on each comparison, with all resulting p-values falling below the 0.05 threshold, signifying statistically significant improvements. This robust statistical evidence firmly establishes CypherViT’s consistent outperformance, bolstering its standing as a superior SSL framework backbone.

**Table 3:** The ablation study’s comparison table details the performance of two SSL families-generative (like MAE) and contrastive (such as SimCLR, MoCo, and Dino)-across different backbones: Vanilla ViT and the newly proposed CypherViT.

Method	CRC(w/ norm)	PanNuke	BreastPathQ	
	Acc$\uparrow$		MSE$\downarrow$	Tau$\uparrow$
MAE^21^	75.06 ± 0.79	74.77 ± 0.66	0.255 ± 0.02	0.228 ± 0.01
SimCLR	(pv: 9.759e−05 < 0.05)	(pv: 3.194e−05 < 0.05)	(pv: 4.785e−05 < 0.05)	(pv: 5.037e−08 < 0.05)
VanillaViT	88.13 ± 0.25	83.06 ± 0.16	0.045 ± 0.003	0.500 ± 0.009
* CypherViT*	**89.47** ± 0.28	**84.85** ± 0.40	**0.025** ± 0.004	**0.640** ± 0.011
MoCo	(pv: 0.0003 < 0.05)	(pv: 0.0008 < 0.05)	(pv: 0.0002 < 0.05)	(pv: 2.537e−05 < 0.05)
VanillaViT	83.63 ± 0.93	81.88 ± 0.57	0.173 ± 0.026	0.317 ± 0.042
* CypherViT*	**86.68** ± 0.40	**83.82** ± 0.47	**0.086** ± 0.005	**0.544** ± 0.032
DINO	(pv: 1.22e−09 < 0.05)	(pv: 4.039e−06 < 0.05)	(pv: 0.005 < 0.05)	(pv: 0.0002 < 0.05)
VanillaViT	90.43 ± 0.14	89.74 ± 0.34	0.036 ± 0.003	0.623 ± 0.018
*CypherViT*	**93.32** ± 0.12	**92.88** ± 0.46	**0.025** ± 0.004	**0.695** ± 0.014

It showcases Top 1 KNN accuracy on the CRC dataset with normalization and the PanNuke dataset, along with MSE and Kendall-Tau scores for BreastPathQ, highlighting CypherViT’s versatility as a model-agnostic, high-performing plug-and-play solution for various SSL frameworks (note: pv refers p-value from t-test).

Significant values are in bold.

## Discussion

For possible future developments, the unique design of our proposed backbone encoder of expanding the class tokens in ViT and aggregating in the final stage has potential beyond serving as a general-purpose feature extractor in two possible extensions. If trained in SSL paradigm, by equipping each histopathological image with a list of domain-specific attributes as supervisory signals for multiple auxiliary tasks (e.g. magnification level, Hematoxylin channel), each class token at the final stage can be customized to predict the individual label for each of the corresponding auxiliary tasks simultaneously. If trained with some supervisory signals as in weakly-supervised settings, each class token at the final stage can be customized to learn targeted lesion regions distinctively.

## Methods

### Overview

In this work, we propose *CypherViT*, cluster-based histopathology phenotype representation learning by self-supervised multi-class-token hierarchical ViT. We incorporate CypherViT into DINO SSL^10^. The entire SSL framework is formulated in Algorithm 1. The detailed architecture of *CypherViT* and its most significant attention module are demonstrated in Figs. 3 and 4, respectively. In this section, we will introduce the *CypherViT* architecture and its advantage for SSL.

### CypherViT architecture

The proposed CypherViT is a novel backbone network in self-supervised learning (SSL) frameworks. It extends the conventional Vision Transformer (ViT) architecture by augmenting its class token system and integrating a sophisticated hierarchical attention mechanism that clusters image patches based on semantic similarity. The attention mechanism within CypherViT operates on a multi-level basis, where each level consists of two critical processes. Initially, a multi-head self-attention block assesses the interrelationships between different patches of the image. Following this, a semantic clustering block groups semantically similar patches together. This hierarchical clustering is meticulously designed to process features from the simplest to the most complex in a bottom-up approach. As the network processes an image, it generates intermediate tokens that encapsulate the aggregated features. These tokens are dynamic and evolve during the learning phase through backpropagation. With each iteration, these enriched tokens serve as input for the subsequent level of the hierarchy, progressively enhancing the feature representation. In the culmination of this hierarchical process, each token becomes a distilled representation of a specific visual concept, capturing essential aspects of histological phenotypes such as cellular structures or stromal patterns as shown in Fig. 3.

To elaborate on the attention module in *CypherViT*, as illustrated in Fig.  4, here, the input is composed of two elements: patch tokens and [cls] token(s). The patch tokens, represented as $\{P_i\}^{N_p}_{i=1}$ where *N* signifies the total number of patches, remain consistent with the standard ViT. Furthermore, CypherViT introduces a significant change by expanding the single [cls] token found in ViT to a set of multiple [cls] tokens, denoted by $\{C_j^{s_k}\}^{N_c}_{j=1}$. The $N_c$ indicates the count of learnable class tokens at a given stage $s_k$, a quantity that is not learned but rather a predefined hyperparameter. The architecture is designed so that at each hierarchical stage $s_k$, the number of these learnable class tokens $N_c$ is reduced. This reduction is based on the understanding that as the network identifies more abstract features, it can represent the data with fewer, more general clusters. For clarity in Fig. 4, certain elements like patch embedding and position encoding are excluded from the visual representation, however, they are indeed part of the actual model. It should be noted that, a stage $s_k$ comprises of a multi-head self-attention block followed by a semantic clustering block, generating an output for the subsequent stage $s_{k+1}$. At the initial stage, the set of patch tokens $\{P_i^{s_k}\}^{N_p}_{i=1}$ and multi-[cls] tokens $\{C_j^{s_k}\}^{N_c}_{j=1}$ are combined to form a concatenated matrix *M*. This matrix, denoted as $M=[\{P_i^{s_0}\}^{N_p}_{i=1}, \{C_j^{s_0}\}^{N_c}_{j=1}]$, is then processed by the multi-head self-attention block. Within this block, the self-attention mechanism operates by transforming the matrix *M* through a series of weight matrices $W_{q_1}$, $W_{k_1}$, and $W_{v_1}$ to generate query ($q_1$), key ($k_1$), and value ($v_1$) components respectively. These components interact in a self-attention calculation $(q_1 k_1^T) v_1 = M_1$. Following this operation, the resulting matrix $M_1$ is divided back into patch tokens $P_1$ and [cls] token set $C_1$. These are then used as inputs for a repeated application of the self-attention mechanism within the semantic-clustering block.

In the module described above, the calculation of attention, denoted as $q k^T$, represents the process of determining the similarity between learnable multi-[cls] tokens *C* and patches *P*, and this process is visually represented by the color orange in Fig. 4. Initially, we showcase this attention mechanism for the first stage only. However, in subsequent stages, the inputs are altered: instead of patches and multi-[cls] tokens, we use multi-[cls] tokens from both the previous stage ($C^{s_{k-1}}$) and the current stage ($C^{s_k}$), where *k* denotes a stage greater than zero. This progression is depicted in the *CypherViT* model illustrated in Fig. 3. The attention at any given stage *k* is mathematically represented as the softmax of the computed similarity matrix as follows:

1 $$\begin{aligned} (q k^T)^{s_k} = Attn^{s_k}_{i, j} = \frac{ \exp ( W_{p} P^{s_k}_i \cdot W_{c} C^{s_k}_{j} + \gamma _j ) }{ \sum _{u=1}^{N_c} \exp ( W_{p} P^{s_k}_i \cdot W_{c} C^{s_k}_{u} + \gamma _u ) } \end{aligned}$$

As the input for the next step, we obtain the learnable multi-[cls] tokens for next stage as:

2 $$\begin{aligned} (q k^T) v = C^{s_{k+1}}_j = C^{s_{k}}_j + W \cdot \frac{ \sum ^{N_p}_{i=1} Attn^{s_k}_{i, j} \cdot W_v P^{s_k}_i }{ \sum ^{N_p}_{i=1} Attn^{s_k}_{i, j} } \end{aligned}$$

The equation includes *W*, $W_p$, $W_c$, and $W_v$, which are all learnable weights within various linear projections. To provide a tangible view of what happens at stage *k*, we interpolate the feature to visualize the attention map, as can be seen on the right side of Fig. 4. Notably, our observations reveal that the post-clustering features, which are derived from learnable multi-[cls] tokens, clearly exhibit attention-focused regions. These regions align with morphologically distinctive tissue phenotypes within histopathological patches. When it comes to computing the loss objective, we utilize an average pooling layer to consolidate the outputs from the semantic-clustering block at the final stage. This step is crucial for achieving a condensed representation that can be effectively used for subsequent analysis or classification tasks.

### SSL framework and training objectives

With the CypherViT backbone established, now we proceed to how the *CypherViT* model is incorporated within the SSL framework. Drawing inspiration from the DINO self-distillation approach, we use the output probabilities from a teacher network as a guiding signal for training a student network. The teacher network’s weights are not directly trained but are instead updated using an exponential moving average (EMA) method during back-propagation. In mathematical terms, the EMA update is represented as $\theta _t \leftarrow \lambda \theta _t + (1 - \theta _t)\theta _s$, where $\theta _t$ denotes the teacher’s weights and $\theta _s$ the student’s weights. The training process involves minimizing the cross-entropy loss, which is calculated by comparing the global and local views generated from the original input image. These views are visual representations augmented from the input image and are crucial in the context of SSL, as depicted in Fig. 3. It’s important to highlight that within the SSL framework, the number of local views and the number of class tokens are independent variables. They are hyperparameters that can be fine-tuned separately, and their individual effects on the model’s performance have been thoroughly investigated through various ablation studies, as shown in Table 4. For clarity, we refer to the global views as $z_g$ and the local views as $z_l$. The objective function for the loss is formulated as:

3 $$\begin{aligned} \mathcal {L}_{CE} = \min _{\theta _s, \psi _s, \phi _s} \sum _{ g \in [1, N_g] } \sum _{ l \in [1, N_l] } -P_{\phi _t} \left[ H_{\psi _t} \left( E_{\theta _t}(x_g) \right) \right] \log P_{\phi _s} \left[ H_{\psi _s} \left( E_{\theta _s}(x_l) \right) \right] . \end{aligned}$$

**Table 4:** Ablation study on two components of our proposed DINO-*CypherViT*, the number of [cls] tokens for feature clustering used at the last stage, and the number of local views used in the multi-crop augmentation strategy.

Ablations		CRC—w/o norm		CRC—w/norm		PanNuke		BreastPathQ	
# of [cls]	# of local views	Acc@1$\uparrow$	Acc@3$\uparrow$	Acc@1$\uparrow$	Acc@3$\uparrow$	Acc@1$\uparrow$	Acc@3$\uparrow$	MSE$\downarrow$	Tau$\uparrow$
4	0	85.80	96.50	91.74	97.24	91.75	**97.13**	0.029	0.65
4	2	87.38	98.06	**93.37**	**99.26**	**93.67**	96.88	0.026	0.66
4	4	**89.05**	**98.90**	92.92	99.13	93.15	96.80	**0.021**	0.69
4	8	87.70	97.10	92.33	97.98	92.28	96.95	0.023	0.69
8	0	85.36	97.03	88.50	98.44	87.65	95.70	0.028	0.64
8	2	85.48	98.41	93.23	98.96	91.75	96.27	0.028	0.63
8	4	86.53	98.27	91.50	98.19	90.51	96.15	0.025	0.66
8	8	86.14	97.57	90.58	97.38	89.87	95.48	0.022	**0.70**

Significant values are in bold.

## Conclusion

In this paper, we propose a **C**luster-based histopathology phenotype representation learning by self-supervised multi-class-token hierarchical ViT (*CypherViT*) as a novel backbone integrated into the SSL paradigm. Through comprehensive experiments on histopathology datasets, we have made a few interesting discoveries. Firstly, on patch-level tasks like tissue type classification and tumor cellularity prediction, our model is proven to outperform existing SSL models qualitatively and quantitatively. Secondly, we find that robustness of SSL features is improved with the optimal number of local views from multi-crop augmentation depending on tasks. Thirdly, in the tested datasets, the utilization of 4 multi-cls tokens at the final stage consistently produced better results than employing 8 cls tokens. Nevertheless, the paper does not thoroughly investigate the correlation between the type of dataset and the required number of multi-cls tokens, leaving an intriguing path for future exploration.

## Data Availability

The datasets used in this study, including the VGH dataset (H &E breast cancer dataset), the CRC dataset (colorectal cancer), the BreastPathQ dataset (invasive breast cancer), and the PanNuke dataset (nuclei instance images), are publicly available. These datasets can be accessed from their respective sources mentioned in the manuscript. Researchers can visit the provided sources to obtain the datasets and utilize them for further analysis and replication of the study findings.
